# Role of miRNAs in Normal and Myasthenia Gravis Thymus

**DOI:** 10.3389/fimmu.2020.01074

**Published:** 2020-06-10

**Authors:** Mélanie A. Cron, Émilie Guillochon, Linda Kusner, Rozen Le Panse

**Affiliations:** ^1^Sorbonne University, INSERM, Association Institute of Myology, Center of Research in Myology, Paris, France; ^2^Department of Pharmacology and Physiology, The George Washington University, Washington, DC, United States

**Keywords:** autoimmunity, thymic involution, germinal center, early-onset myasthenia gravis, thymoma, thymocytes, thymic epithelial cells

## Abstract

The thymus, a primary lymphoid organ, provides a complex environment essential for the generation of the T-cell repertoire. Thymic alterations occur during life either in the context of thymic involution upon aging or the pathophysiological context of Myasthenia Gravis (MG). These changes involve complicated regulatory networks, in which microRNAs (miRNAs) are key players. Here, we analyzed the role of miRNAs in thymocyte maturation and differentiation sustained by thymic epithelial cells. We compared data from the literature regarding the role of mouse thymic miRNAs and original data obtained from a human thymic miRnome study. We identified a set of highly expressed miRNAs defined as ThymiRs and investigated miRNA expression in infants as compared to adults to determine those associated with human thymic involution. Thymic changes are also frequently observed in MG, an autoimmune disease which results in the production of anti-acetylcholine receptor (AChR) antibodies that lead to muscle weaknesses. Alterations such as thymoma in late-onset MG patients and hyperplasia with ectopic germinal centers (GCs) in early-onset (EOMG) patients are found. Thymic miRNA expression has been studied in AChR-MG patients both in thymoma-associated MG (TAMG) and EOMG, and their function through their mRNA targets investigated. Most of the dysregulated thymic miRNAs in EOMG are associated with GC development, such as miR-7, miR-24, miR-139, miR-143, miR-145, miR-146, miR-150, miR-452, miR-548 or thymic inflammation, such as miR-125b, miR-146, or miR-29. Understanding these pathways may provide therapeutic targets or biomarkers of disease manifestations.

## Overview of miRNAs

MicroRNAs (miRNAs) correspond to non-coding short single-stranded RNAs (~22 nucleotides) that serve as post-transcriptional regulators. They are mainly transcribed and process through the canonical pathway as pri-miRNAs, cleaved into pre-miRNAs by DROSHA and DGCR8 (DiGeorge syndrome Critical Region gene 8) in the nucleus and exported to the cytoplasm by the protein exportin 5. Next, pre-miRNAs are cleaved by DICER and its partner TRBP1 into mature miRNAs. Mature miRNAs coupled with the RNA-induced silencing complex (RISC, a heterogeneous molecular complex) target mRNAs, leading to their degradation or the inhibition of their translation, according to the perfect or imperfect miRNA-mRNA matching, respectively (1). They are involved in physiological and pathophysiological processes, including autoimmune diseases (2).

Autoimmune diseases result from the dysfunction of the immune process. The breakdown of immunological tolerance leads to the presence of autoreactive immune cells which cause the destruction of self (3). miRNAs play crucial roles in the immune process through the development of the immune system, proliferation of key immunological cells, differentiation of cells into their lineage, and apoptosis at immunological checkpoints (4). Disturbance along the process by altered expression of miRNAs and their downstream function can initiate or maintain autoimmune conditions. Involvement of miRNAs, specifically key miRNAs (e.g. miR-21, miR-146, miR-155, miR-146, 125a-5p) is already well-documented in major autoimmune diseases such as lupus erythematosus, multiple sclerosis, diabetes or rheumatoid arthritis. Here, we highlight the significance of miRNAs in the development of the thymus and the ability for dysfunction to result in an autoimmune disease, myasthenia gravis.

## Physiological Role of the Thymus

The thymus provides a complex environment essential for the generation of the T-cell repertoire. It is composed of various cell types, essentially thymocytes and thymic epithelial cells (TECs), but also fibroblasts, myoid cells, dendritic cells, macrophages, and B cells. Differentiation of thymocytes occurs through interactions with stromal cells while they are progressing in the different thymus compartments (5).

In their first differentiation steps, immature thymocyte precursors become progressively double positive (DP) for CD4 and CD8 co-receptors and acquire a complete T-cell receptor (TCR). Further successful differentiation of thymocytes depends on the quality and the specificity of the interaction of their T-cell receptor (TCR) with self-major histocompatibility complex (MHC) molecules. The large majority of thymocytes die either because the TCR-MHC interaction is too weak (death by neglect) or, in contrast, because the TCR-MHC interaction is too strong (negative selection, self-tolerance). In parallel, positively selected CD4^+^CD8^+^ thymocytes end up single positive (SP) for either CD4 or CD8 (lineage commitment) (5). Only a few thymocytes pass successfully selection and are exported to the periphery where they will differentiate into different T-cell subsets. However, within the thymic environment, some T cells can differentiate into natural regulatory T (Treg) cells (5).

TECs represent the main cell type amongst thymic stromal cells and include cortical and medullary TECs (cTECs and mTECs). mTECs play a central role in negative selection of thymocytes through their capacity to express a wide range of tissue-specific antigens (TSAs) and mediate depletion of autoreactive T cells. The ectopic expression of TSAs by mTECs is controlled by epigenetic factors and by transcription factors, the most well-known being the autoimmune regulator (AIRE) (6). The thymus is fully active during the neonatal period and undergoes an involution process early during life (after 1-year-old). Thymic involution is characterized by the alteration of thymic architecture and the loss of thymic function. In particular, involution is associated with a decrease in TECs, more prominent for mTECs as compared to cTECs, and replacement by fat tissue. Despite the decrease in cellular density, the adult thymus still contains thymocytes and maintains the proportion of the principal thymocyte subsets indicating that the thymus remains active during adult life (7).

## Implications of miRNAs in the Mouse and Human Thymus

The role of enzymes involved in the biogenesis of miRNAs and specific miRNAs in T-cell lineage in the periphery has been largely documented (8, 9). Here, we will specifically review the role of miRNAs in the thymus, in particular in thymic epithelial cells (TECs) with their potential impact on thymic architecture, and on thymocyte differentiation.

**Figure 1:**
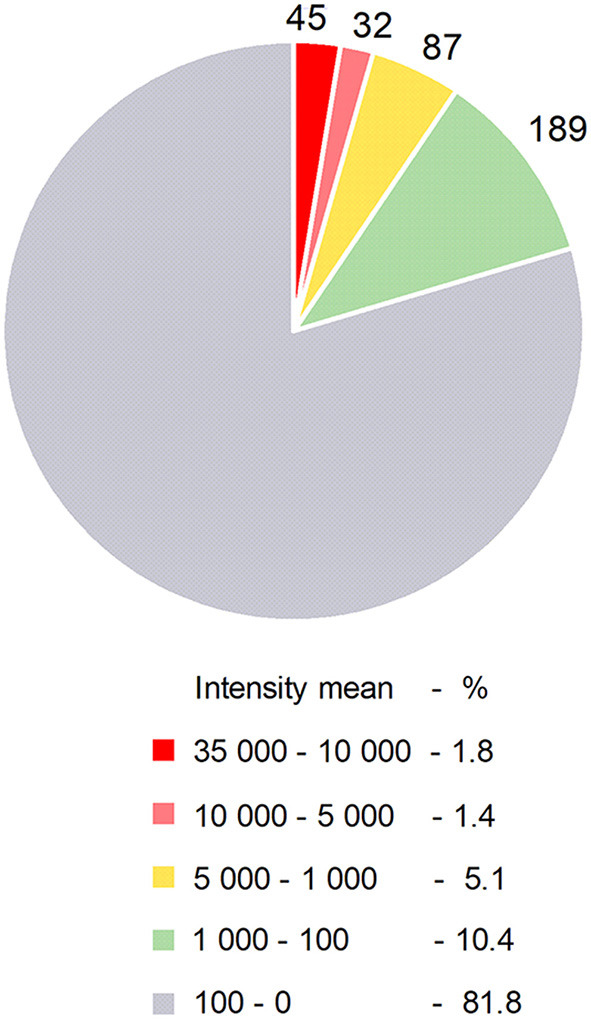
Human ThymiRs. Repartition of ThymiRs according to the mean of intensities. Thymic biopsies were collected from female infants undergoing cardiovascular surgery at the Marie Lannelongue Surgical Center (Le Plessis-Robinson, France) (*n* = 6, 3–12 months old). Studies on thymuses were approved by a local ethics committee (CPP, authorization number ID RCB 2010-A00250-39). Total RNA extraction and miRNA analyses were done as described in Cron et al. (11). Raw data were imported using R package pd.mirna.3.0 (v 3.12.0). Raw intensity values were background corrected and normalized with RMA (Robust Multi-array Average) function from oligo R package (1.48.0).

Certain miRNAs are defined as immuno-miRs as they regulate many functions related to the immune system, or myomiRs, which are more particularly expressed in skeletal muscle. The most well-known immuno-miR are miR-146, miR-155 and the cluster miR-17~92 (10). From original results detailed in Figure 1 and Table 1, we analyzed the human thymus of infants for the most expressed miRNAs that could be defined as ThymiRs (Figure 1). The highly expressed human ThymiRs contained the most well-known immuno-miR, such as miR-146, miR-150, miR-155, miR-181 subtypes, some miRNAs from the miR-17~92 cluster and the paralogous clusters miR-106b-25 and miR-106a-363. They also included six let-7 subtypes (Table 1).

**Table 1:** Human ThymiRs.

**Name**	**Intensity mean**	**SEM**
hsa-miR-16	42,177	995
hsa-miR-181a	39,264	987
hsa-miR-26a	36,825	751
hsa-miR-17	32,468	245
hsa-miR-106a	30,695	134
hsa-miR-20a	29,018	241
hsa-miR-3960	28,997	3,351
hsa-miR-3665	26,827	2,385
hsa-miR-92a	26,734	560
hsa-miR-181b	26,425	657
hsa-miR-103a	23,522	459
hsa-miR-4787-5p	23,473	2,736
hsa-miR-342-3p	23,080	453
hsa-let-7a	22,697	1,680
hsa-let-7b	22,524	1,506
hsa-miR-93	21,822	810
hsa-miR-191	21,059	726
hsa-let-7c	20,657	573
hsa-miR-205	20,509	806
hsa-miR-107	20,000	367
hsa-miR-125b	19,037	722
hsa-miR-106b	17,449	652
hsa-let-7d	16,420	412
hsa-miR-4497	16,199	903
hsa-miR-20b	15,256	473
hsa-miR-150	15,058	415
hsa-miR-19b	14,736	1,521
hsa-miR-155	14,724	401
hsa-miR-4466	14,691	1,656
hsa-miR-1915	13,850	1,881
hsa-miR-4488	13,612	1,872
hsa-miR-638	13,379	1,563
hsa-miR-24	13,138	957
hsa-miR-15b	12,644	1,160
hsa-miR-23a	12,537	170
hsa-let-7i	12,007	1,014
hsa-miR-3656	11,829	2,541
hsa-miR-320b	11,481	1,282
hsa-miR-320a	11,370	1,221
hsa-miR-2861	10,996	1,367
hsa-miR-100	10,871	313
hsa-miR-200c	10,783	630
hsa-miR-15a	10,574	1,133
hsa-miR-4516	10,522	1,110
hsa-miR-320c	10,430	1,061

*List of the most highly expressed ThymiRs with a mean intensity over 10,000. The origin of data is detailed in Figure 1. Raw data were imported using R package pd.mirna.3.0 (v 3.12.0). Raw intensity values were background corrected and normalized with RMA (Robust Multi-array Average) function from oligo R package (1.48.0). Intensity means and standard error of the mean (SEM) were calculated from the normalized intensity values obtained using Limma R package (v 3.40.6)*.

### miRNAs and Thymic Epithelial Cells

Different studies have proved that molecules involved in the biogenesis of miRNAs play a central role in TEC differentiation.

Using *FoxN1 (Forkhead box protein N1)*-Cre knock-in mice to conditionally inactivate *Dgcr8* in TECs, Khan et al. demonstrated that *Dgcr8* is critical for maintaining a proper thymic architecture and that canonical miRNAs are required to support TEC cellularity and differentiation. In particular, they observed a progressive loss of AIRE^+^ mTECs that could affect central tolerance and favor the development of autoimmune diseases (12). Embryonic loss of *Dicer* in TECs results in premature thymic involution, progressive disorganization of the thymic epithelium and the formation of epithelial voids. The normal differentiation and function of TECs are altered, impacting thymocyte development and inducing phenotypic changes in peripheral T cells. Loss of *Dicer* expression in TECs clearly affects T-cell development from the second week of life with an increase in DP and a decrease in CD8^+^ and CD4^+^ SP T cells (13). A higher number of double-negative (DN) cells is also observed, in part due to an increased number of immature B cells in the thymus of these *Dicer* deficient mice. They could develop *in situ*, possibly due to changes in the microenvironment as the thymic medulla progressively displayed more Lyve-1 lymphatic vessels, PNAd high endothelial venules, and CR1 follicular dendritic cells that are normally observed in secondary lymphoid tissues (13, 14). Besides, several publications showed a decrease in both *Aire*-dependent and -independent TSAs in *Dicer*-deficient mTECs (15); altered expression of TSAs in mice that lack *Dicer* expression in TECs could be correlated with multiorgan infiltrations (13).

Papadopoulou et al. demonstrated that certain features of the premature thymic involution phenotype of *Dicer* mutants are recapitulated in mouse mutants lacking *miR-29a*; however, these animals do not display the same architectural disruption of the thymus, implying that other miRNAs regulate TEC maintenance (14). Besides, the absence of *miR-29a* selectively affects the *Aire*-dependent TSA gene expression (15). However, an increase in thymic *miR-29* subtypes is observed together with thymic involution in aging mice (16).

The changes observed in *Dicer* or *Dgcr8* deficient mice are usually attributed to the loss of miRNAs. However, we have to keep in mind that DICER can process other types of RNAs and regulate different cellular functions beyond its endonuclease activity (17). As for DGCR8, it is involved in maintaining heterochromatin organization and attenuating senescence, independently of its microRNA-processing activity (18).

Ucar et al. analyzed the expression of miRNAs in isolated cTECs and mTECs in human and mouse thymuses. They demonstrated that certain miRNAs are differentially expressed in cTECs and mTECs, and even differentially expressed upon mTEC maturation. Using different approaches for selected miRNAs they observed that in mice some miRNAs are down-regulated in CD80^+^AIRE^+^mTECs as compared to CD80^+^AIRE^−^mTECs. This suggests that TEC differentiation could be associated with a decreased expression of certain miRNAs allowing, for example, a higher expression of AIRE. Inversely, AIRE regulates the expression of specific miRNAs. They showed in AIRE-deficient mice that some miRNAs can be either up or down-regulated in CD80^+^mTECs as compared to wild type mice (15). This was confirmed by Macedo et al. that demonstrated that silencing *Aire* in mouse mTECs leads to the up- and down-regulation of specific miRNAs (19). The identified dysregulated miRNAs from these studies were different. However, we can hypothesize that in AIRE^+^TECs, miRNAs that are decreased could lead to the specific expression of TSAs that are implicated in central tolerance mechanisms.

### miRNAs and Thymocyte Development

*Dicer* deletion from the double-positive stage of T-cell development compromises the survival of αβ lineage cells and results in a decreased thymic cellularity in DP and SP T cells. Surprisingly, *Dicer* seems to be dispensable for CD4^+^ and CD8^+^ T cell lineage commitment (20, 21). *Dicer* or *Drosha* deletion at a later stage in CD4^+^ T cell does not alter the number and composition of thymocytes, though it results in a reduction in thymic CD4^+^CD25^+^Foxp3^+^ natural Treg cells. *Dicer* depletion in CD4^+^ thymocytes also results in the reduction of invariant natural killer T (iNKT) cells (22). Besides, mice with T-cell specific *Dicer* or *Drosha* deficiency develop immune pathologies, in particular, inflammatory bowel disease (23) and organ inflammation (24). Deletion of *Dicer* or *Drosha* results in the loss of mature miRNAs generated via the canonical biogenesis pathways in which not all miRNAs are deleted since other biogenesis pathways are involved (1). Nevertheless, these results demonstrate that miRNAs can modulate T-cell development. Different studies have shown that individual miRNAs are dynamically regulated during T-cell development and maturation as detailed below (25–27).

### Implication of miRNAs in Thymic Involution

Thymic involution is a natural process occurring with age and an adaptive process in response to stress situations. Thymic involution is characterized by morphological and functional changes and includes TEC-driven programmed thymic involution and thymocyte apoptosis. As miRNAs regulate numerous physiological and pathophysiological processes, they are probably key orchestrators of thymic involution.

#### Thymic Involution With Aging

The expression of miRNAs is clearly modified in the aging mouse thymus (16). Guo et al. investigated thymic miRNA expression in 3 and 12 months old female and male mice as thymic involution is often described as being also sex-hormone dependent. Thymic atrophy is clearly observed in the first year of life, and even more in males as compared to females. Specific miRNAs exhibit age- or sex-differentially expression. Among them, *miR-2137* is increased in the aging thymus and particularly in mTECs (28). Some miRNAs seem dysregulated more specifically in female mice and to determine if the female-biased miRNAs were linked to estrogen, the expression of specific miRNAs was measured in the thymus either in ovariectomized or castrated mice treated with estradiol. Results show that *miR-27b* and *miR-378a* are estrogen-responsive miRNAs in mouse thymus (28).

miRNAs expression has also been investigated in isolated TECs from aging-mice and 17 miRNAs were showed to be down-regulated upon aging. In particular, miRNAs known to possess immune function, such as *miR-146, miR-150, miR 155, miR-181* subtypes and some miRNAs from the *miR-17*~*92* cluster. The decreases observed in TECs are correlated with the age-related thymic weight loss suggesting that miRNA decreased expression in TECs precedes thymic involution (29).

In human thymus, we analyzed dysregulated thymic miRNAs in female adults (15–33 years old) compared to female infants (3–12 months old). Of the miRNAs assessed, 56 were up-regulated and 87 were down-regulated in the adult thymuses upon aging, respectively (Tables 2A,B). From these lists of dysregulated miRNAs in the course of thymic involution, among the up-regulated ones, those that seemed particularly interesting were those that reached in adults a fluorescence intensity mean above 1,000 (arbitrarily chosen). These results must be taken into account with caution due to the small sample size and the lack of validation. However, several of them, such as let-7b, miR-139, miR-193a, miR-214, miR-27b are also up-regulated in the thymus of male mice upon aging (28). miR-182 and miR-200b are also found up-regulated in isolated TECs from aging mice (30). miR-195a-5p is highly up-regulated in the TECs isolated from the aging mice inhibiting the proliferation of mTECs by directly targeting Smad7 (31). As for miR-451, its expression is up-regulated in the thymus of systemic lupus erythematosus mouse (32).

**Table 2:** Dysregulated miRNAs during human thymic involution.

**miRNA ID**	**Intensity mean**		**FC**	***p*-value**
	**Infants**	**Adults**		
**(A) Up-regulated miRNAs in adults vs. infants (FC** **≥** **1.5**, ***p*** **≤** **0.05)**				
hsa-miR-383	9	182	20.32	9.88E-06
**hsa-miR-486-5p**	337	1,475	4.38	6.59E-03
**hsa-miR-451**	485	1,489	3.07	1.93E-02
hsa-miR-486-3p	14	41	3.01	4.25E-03
**hsa-miR-3609**	565	1,684	2.98	1.68E-03
hsa-miR-34c-3p	105	284	2.72	1.97E-03
**hsa-miR-214**	871	2,292	2.63	2.29E-02
hsa-miR-375	124	309	2.48	1.64E-03
**hsa-miR-206**	749	1,794	2.39	1.85E-02
hsa-miR-494	350	831	2.37	1.42E-02
hsa-miR-139-3p	35	82	2.32	7.48E-03
**hsa-miR-149**	865	2,000	2.31	7.31E-04
hsa-miR-134	69	158	2.29	9.41E-03
hsa-miR-650	16	37	2.26	1.99E-02
hsa-miR-224*	72	160	2.21	3.13E-03
hsa-miR-139-5p	356	781	2.20	1.14E-03
hsa-miR-30c-2*	10	22	2.17	6.75E-03
**hsa-miR-182**	2,116	4,534	2.14	5.98E-03
hsa-miR-143*	27	57	2.09	1.45E-02
hsa-miR-3605-5p	17	34	2.07	2.79E-03
hsa-miR-664*	141	291	2.06	1.62E-03
**hsa-miR-195**	4,561	9,012	1.98	4.33E-05
hsa-miR-4788	18	35	1.94	3.03E-04
hsa-miR-572	145	280	1.93	5.30E-03
hsa-miR-10b	308	593	1.93	5.24E-03
hsa-miR-3607-5p	102	197	1.92	6.24E-04
hsa-miR-584	10	19	1.90	2.08E-02
hsa-miR-34c-5p	132	247	1.87	3.53E-03
**hsa-miR-145**	7,014	13,024	1.86	1.68E-03
hsa-miR-125a-3p	52	96	1.84	2.21E-02
hsa-miR-4496	8	15	1.83	2.13E-02
hsa-miR-193a-5p	357	650	1.82	1.15E-02
**hsa-let-7e**	3,651	6,563	1.80	1.50E-02
**hsa-let-7b**	22,524	39,436	1.75	6.66E-05
hsa-miR-200b*	449	784	1.75	2.44E-03
hsa-miR-30a*	90	155	1.73	1.30E-02
hsa-miR-492	7	12	1.73	4.09E-02
hsa-miR-4324	36	62	1.72	4.38E-02
hsa-miR-4657	10	17	1.71	4.90E-02
**hsa-miR-455-3p**	3,410	5,800	1.70	3.35E-02
hsa-miR-4778-5p	7	12	1.70	9.78E-03
hsa-miR-1202	78	133	1.69	3.37E-02
hsa-miR-4800-5p	25	42	1.67	1.95E-02
hsa-miR-379	180	299	1.66	1.91E-02
**hsa-miR-4530**	3,746	6,216	1.66	1.96E-02
hsa-miR-4738-3p	32	54	1.66	2.33E-03
hsa-miR-4667-5p	56	92	1.64	2.81E-03
hsa-miR-29b-1*	56	90	1.61	1.17E-02
hsa-miR-23b*	16	26	1.60	4.04E-02
**hsa-miR-34a**	1,415	2,225	1.57	9.55E-04
hsa-miR-1	6	9	1.57	1.32E-03
hsa-miR-339-3p	409	636	1.56	4.02E-02
**hsa-miR-27b**	1,818	2,825	1.55	1.94E-02
**hsa-miR-143**	5,850	9,013	1.54	2.24E-02
hsa-miR-3127-5p	8	12	1.53	2.77E-02
**hsa-miR-4505**	867	1,311	1.51	4.28E-02
**(B) Down-regulated miRNAs in adults vs. infants (FC** **≤−1.5**, ***p*** **≤** **0.05)**				
hsa-miR-301a	310	55	−5.62	9.13E-03
hsa-miR-449b	70	12	−5.61	1.03E-03
hsa-miR-142-3p	80	15	−5.30	8.44E-04
hsa-miR-449a	70	14	−4.90	1.65E-03
hsa-miR-142-5p	269	68	−3.94	4.59E-03
hsa-miR-297	69	19	−3.57	4.02E-02
hsa-miR-4793-3p	76	22	−3.41	4.74E-02
hsa-miR-551a	32	10	−3.18	7.07E-04
hsa-miR-590-5p	36	12	−2.96	4.83E-02
hsa-miR-15a*	52	18	−2.91	3.14E-04
hsa-miR-424	56	20	−2.86	5.79E-03
hsa-miR-502-5p	32	12	−2.71	6.54E-03
hsa-miR-3907	24	9	−2.71	7.61E-03
hsa-miR-598	16	6	−2.62	7.05E-03
hsa-miR-550a*	113	43	−2.61	6.50E-04
hsa-miR-542-5p	139	54	−2.55	6.75E-03
**hsa-miR-19a**	1,019	406	−2.51	3.46E-03
hsa-miR-19b-1*	40	16	−2.49	1.38E-02
hsa-miR-449c	37	15	−2.48	4.13E-03
hsa-miR-641	143	58	−2.48	5.63E-04
hsa-miR-4440	139	59	−2.37	3.05E-02
hsa-miR-301b	10	4	−2.29	4.94E-03
**hsa-miR-18b**	1,711	752	−2.28	6.44E-06
**hsa-miR-15a**	10,574	4,666	−2.27	1.66E-03
hsa-miR-140-5p	773	342	−2.26	7.22E-03
hsa-miR-3201	21	10	−2.21	1.42E-02
**hsa-miR-141**	1,840	838	−2.20	4.19E-03
hsa-miR-181a*	2,719	1,239	−2.19	4.93E-05
hsa-let-7g*	62	28	−2.17	3.14E-02
hsa-miR-16-1*	8	4	−2.16	3.51E-03
hsa-miR-3064-3p	57	27	−2.15	1.02E-03
hsa-miR-589	15	7	−2.14	1.68E-02
hsa-miR-503	405	190	−2.13	1.04E-02
hsa-miR-122	67	31	−2.13	3.20E-03
hsa-miR-4286	147	70	−2.10	1.52E-02
hsa-miR-5096	27	13	−2.09	3.94E-02
hsa-miR-362-3p	22	11	−2.09	3.72E-02
hsa-miR-431	17	8	−2.05	8.44E-03
hsa-miR-4786-5p	37	18	−2.03	4.76E-02
hsa-miR-330-5p	17	8	−2.00	6.59E-03
hsa-miR-3620	29	15	−1.99	5.08E-03
hsa-miR-185*	25	13	−1.94	2.05E-02
hsa-miR-4787-3p	19	10	−1.93	1.41E-02
hsa-miR-196b*	264	137	−1.92	2.52E-03
hsa-miR-484	216	114	−1.90	1.71E-04
hsa-miR-1306	24	13	−1.90	1.37E-02
hsa-miR-637	45	24	−1.89	4.67E-02
hsa-miR-551b	22	11	−1.88	4.24E-02
hsa-miR-612	18	10	−1.88	8.73E-03
hsa-let-7d*	35	18	−1.87	1.06E-02
hsa-let-7i*	67	36	−1.85	2.79E-03
hsa-miR-1915*	39	21	−1.83	4.31E-02
hsa-miR-3157-5p	37	20	−1.82	4.62E-03
**hsa-miR-128**	6,129	3,418	−1.79	7.24E-03
**hsa-miR-181d**	2,103	1,178	−1.79	4.14E-03
hsa-miR-130b*	12	7	−1.77	2.13E-02
**hsa-miR-19b**	14,736	8,370	−1.76	1.26E-02
**hsa-miR-210**	1,280	731	−1.75	1.82E-02
hsa-miR-129-5p	13	8	−1.74	3.28E-02
hsa-miR-760	25	14	−1.74	4.16E-02
hsa-miR-4493	6	4	−1.73	1.87E-02
hsa-miR-1229	13	8	−1.72	1.76E-03
**hsa-miR-363**	4,120	2,399	−1.72	3.07E-02
hsa-miR-4763-5p	34	20	−1.72	9.15E-03
**hsa-miR-18a**	6,309	3,714	−1.70	9.64E-03
**hsa-miR-30e**	2,171	1,282	−1.69	1.91E-02
hsa-miR-4323	31	18	−1.69	5.58E-03
hsa-miR-4746-3p	15	9	−1.67	1.97E-02
**hsa-miR-106b**	17,449	10,554	−1.65	4.93E-03
hsa-miR-3928	33	20	−1.65	1.48E-02
hsa-miR-675*	15	9	−1.64	9.47E-03
hsa-miR-4284	262	163	−1.61	1.46E-02
hsa-miR-3610	19	12	−1.61	1.72E-02
hsa-miR-4457	8	5	−1.60	2.84E-02
hsa-miR-361-3p	78	49	−1.60	4.66E-02
hsa-miR-18a*	375	236	−1.59	1.60E-03
**hsa-miR-130b**	7,565	4,770	−1.59	3.91E-02
hsa-miR-4478	42	27	−1.58	4.53E-02
hsa-miR-3180	438	280	−1.57	3.58E-02
hsa-miR-17*	1,384	890	−1.56	1.75E-03
**hsa-miR-130a**	5,672	3,709	−1.53	2.13E-02
hsa-miR-20b*	229	150	−1.52	9.11E-03
hsa-miR-636	20	13	−1.52	2.70E-02
hsa-miR-3145-5p	29	19	−1.52	1.97E-02
hsa-miR-1304	12	8	−1.51	1.19E-02
hsa-miR-769-5p	285	188	−1.51	2.86E-03
hsa-miR-1226	10	7	−1.51	2.61E-02

*Thymic biopsies were collected from female donors undergoing cardiovascular surgery at the Marie Lannelongue Surgical Center (Le Plessis-Robinson, France): infants (n = 6, 3–12 months old) and adults (n = 6, 15-33 years old). Studies on thymuses were approved by a local ethics committee (CPP, authorization number ID RCB 2010-A00250-39). Total RNA extraction was done as described in Cron et al. (11). miRNA expression was measured using the Affymetrix GeneChip miRNA 3.0 Array (Santa Clara, USA) by the Genopolis consortium (Milano, Italy)*.

*Raw data were imported using R package pd.mirna.3.0 (v 3.12.0). Raw intensity values were background corrected and normalized with RMA (Robust Multi-array Average) function from oligo R package (1.48.0). Differential expression analysis was performed using Limma R package (v 3.40.6). Limma fits a linear model to expression data for each miRNA and Empirical Bayes method was used to generate differential expression statistics. The tables list mature miRNAs up- (A) or down-(B) regulated in adults as compared to infants with a fold change (FC) set at 1.5 and a p < = 0.05. In bold miRNAs of particular interest regarding their mean intensity value. (A), miRNAs at least above 1,000 in adults. (B), miRNAs at least above 1,000 in infants. The mean intensity values are coded as follow: 40,000–10,000 (red), 10,000–5,000 (pink), 5,000–1,000 (orange), 1,000–100 (green), below 100 (gray)*.

**Figure 2:**
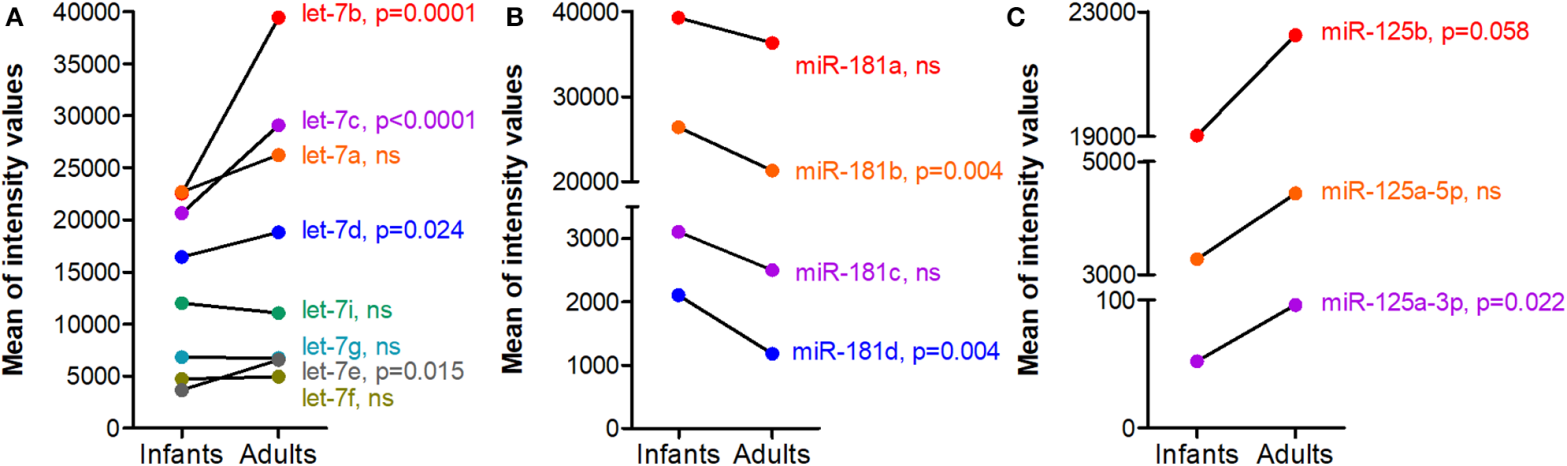
Dysregulated miRNAs during human thymic involution. Thymic biopsies were collected and processed for miRNA analyses as detailed in Figure 1. Mean of normalized intensity values were calculated for each miRNA for infants (*n* = 6, 3–12 months old females) and adults (*n* = 6, 15–33 years old females) data. **(A)** let-7, **(B)** miR-181, and **(C)** miR-125 families. Differential expression analysis was performed using Limma R package (v 3.40.6). Limma fits a linear model to expression data for each miRNA and Empirical Bayes method was used to generate differential expression statistics: *p*-values and fold-change values.

For the down-regulated miRNAs (Table 2B), we observed that miR-181 subtypes are significantly down-regulated with aging (see the miR-181 paragraph and Figure 2A below for more details). Besides, among miRNAs that had a fluorescence intensity mean above 1,000 in infants and that significantly decreased with aging, the majority of them belong to the miR-17~92 cluster or the paralogous miR-106a~363 cluster (33).

#### Thymic Involution Linked to Stress

Thymic involution is also observed upon pathogen infections. This could be mediated via the increased expression of interferon (IFN)-I subtypes that will target thymic different cells. Loss of the miRNAs through the deletion of *Dicer* or the *miR-29a* cluster in TECs results in elevated sensitivity of TECs to Poly(I:C), a molecule mimicking dsRNA from viral infection, and premature thymic involution. In mice, *miR-29a* regulates the expression of IFNAR1 (one of the subunits of the IFN-I receptor) and consequently controls the ability of TECs to respond to pathogen infections and IFN-I signalization (14). Linhares-Lacerda et al. observed the up-regulation of miRNAs in mouse TECs upon *Trypaosoma cruzi* infection but not that of *miR-29* subtypes (34).

Glucocorticoids can also induce thymic involution, mainly by triggering DP T cell apoptosis. Upon thymocytes exposure to glucocorticoids, a decrease expression of *Drosha, Dicer*, and *Dgcr8* is observed and subsequently a decreased expression of miRNAs such as the miRNAs of the *miR-17*~*92* cluster (35, 36). Smith et al. demonstrated that *Dicer* depletion induces T-cell apoptosis while the overexpression of the *miR-17*~*92* cluster reduces it (35).

### Focus on miRNAs Clearly Involved in the Thymic Functions

#### Let-7 miRNAs

The let-7 miRNAs were among the first to be identified in mammals and they represent the most abundant miRNAs in the genome. The let-7 miRNAs consists of 12–14 members encoded on different chromosomes. They contain the identical seed region interacting with mRNAs. Mature functional let-7 miRNA expression is inhibited by the RNA binding protein LIN28 expressed in hematopoietic stem cells (37). An increase of several *let-7* family members is observed together with thymic involution in the thymus of aging mice (16). An increased expression was also observed for numerous let-7 members in female human thymus of adults as compared to infants (Figure 2A).

Pobezinsky et al. investigated the role of *let-7* miRNAs in mouse thymic T-cell differentiation. They demonstrated that *let-7* miRNAs control the expression of the transcription factor Plzf (promyelocytic leukemia zinc finger) that regulates NKT-cell differentiation (38). Recently Xiao et al. demonstrated the role played by *let-7* miRNAs in the limitation of the number of B cells within the thymus. They used a mouse model, designated as Foxn1lacZ, in which the expression of the critical TEC-specific transcription factor *Foxn1* is normal at fetal stages, but down-regulated beginning at postnatal day 7, causing progressive reduction of total thymocytes and premature thymic involution. Early in life in *Foxn1* deficient mice, TECs control the up-regulation of *let-7b* and *let-7g* in hematopoietic stem cells, suppressing *Arid3a* expression in intrathymic B cell progenitors to limit their proliferation during the neonatal to adult transition (39).

In human, let-7a expression is highly up-regulated in mature peripheral CD4^+^ and CD8^+^ T lymphocytes compared to DP thymocytes. Microarray analyses showed the up-regulation of let7-e, let7-g and let-7f in CD4^+^ and CD8^+^ SP thymocytes compared to DP thymocytes and a differential expression for let-7e between CD8^+^ and CD4^+^ SP thymocytes (26).

#### miR-181a

miR-181a is a member of miR-181 family that includes 6 miRNAs: miR-181a-1, miR-181a-2, miR-181b-1, miR-181b-2, miR-181c, and miR-181d (miR-181a-1 and miR-181a-2, as well as miR-181b-1 and miR-181b-2 being identical). In mice, *miR-181a* is highly expressed in DP thymocytes and controls the development of early thymocytes by targeting *TCR* and *CD69* (40, 41). Besides, *miR-181a* deletion impairs the development of NKT cells (42). Li et al. suggest that *miR-181a* acts as an intrinsic antigen sensitivity “rheostat” during T-cell development (40). Thymic *miR-181a* plays a central role in central tolerance and is important for the elimination of autoreactive T cells (41). In the *miR-181* family, *miR-181d* is one of the most stress-responsive miRNA identified in the thymus upon LPS injections. However, the different *miR-181* subtypes could have overlapping or compensatory functions, at least in response to stress (36). In human, Ghisi et al. showed that miR-181d and miR-181c were strongly down-regulated in CD4^+^ as compared to CD8^+^ SP thymocytes (26).

The expression of *miR-181a* and *miR-181b* is down-regulated in old mice compared to young ones (16) and all the miR-181 subtypes are also decreased in female human thymus of adults as compared to infants (Figure 2B). If *miR181a* deficiency disrupts thymocytes development, Stefanski et al. demonstrated that *miR-181a1* and *miR-181b1* are not required for maintenance of thymus integrity and that *miR-181a1* and *miR-181b1* are dispensable for TEC differentiation. They control thymocyte development and mature T-cell export and homeostasis within the periphery (43). However, Guo et al. showed that *miR-181a* is decreased in mTECs from aging mice in link with thymic involution. By transfecting mTECs (MTEC1) with a *miR-181a* mimic, they observed that it induces mTEC proliferation while its antagomiR reversed this effect. *miR-181a* was shown to target transforming growth factor β receptor (*Tgfbr1*). *Tgfbr1* expression increases with age in mice, which is consistent with the decreased level of *miR-181a* in addition to the ability of TGF-β to decrease the proliferation of mTECs (44). Recently, Cotrim-Sousa et al. suggest that *miR181b* and *miR-30b* modulate the expression of adhesion molecules involved in mTEC and thymocyte interactions (45).

#### miR-150

First described in 2007 by Zhou et al. in hematopoietic stem cells (46), miR-150 is described as an immuno-miR regulating immune functions, such as proliferation, apoptosis and differentiation of NK, T and B cells. The role of miR-150 has especially being studied in peripheral blood cells.

In the thymus of mice, two different studies showed that *miR-150* expression decreases in mTECs from aging mice in link with thymic involution (28, 29). *miR-150* differentially controls the development of NK and iNKT cell lineages by targeting *c-Myb* (47). In transgenic mice overexpressing *miR-150*, the development of thymocytes is partially blocked, especially the differentiation of DN3 to DN4, which ultimately leads to a decrease in the number of CD4^+^ and CD8^+^ SP thymocytes (48). An increased expression of *miR-150* is observed during the maturation of iNKT cells in the mouse thymus and *miR-150* deletion results in an interruption of iNKT cell final maturation in both thymus and periphery (49).

From transcriptomic analyses on purified human or mouse thymocytes and TECs, it was observed that miR-150 is highly expressed in thymocytes compared to mTECs or cTECs (15). In the human thymus, Ghisi et al. showed that miR-150 is upregulated as the maturation of T cell progresses. They showed that miR-150 targets NOTCH3, known to be important for T-cell differentiation, and that forced expression of miR-150 reduces NOTCH3 levels in T-cell lines affecting their proliferation and survival (26).

#### miR-146

miR-146 family consists of two miRNAs with nearly identical sequences, miR-146a and miR-146b. They are potent immune-miRs and their role in peripheral blood cells has been largely investigated (50).

In the mouse thymus, *miR-146a* expression fluctuates in thymocytes at different developmental stages, increasing in CD4^+^ and CD8^+^ SP thymocytes compared DP thymocytes (51). The overexpression of *miR-146a* in transgenic mice impairs the process of positive selection during T-cell development and inhibits the further differentiation of selected SP (especially CD8^+^ SP) thymocytes. The authors further identified 9 positive selection-associated genes, which are downregulated in *miR-146a* transgenic mice, such as genes encoding major histocompatibility complex class I/II molecules, *IL-7* receptor α chain, and *Gimap4* (52). However, in transgenic mice with a specific CD4^+^-driven T cell overexpression, T-cell development occurs normally in the *mir-146a* transgenic mice and a decrease of CD4^+^ T-cells is observed together with a reduction in surface expression of CD62L, which is normally upregulated upon thymic egress. Besides, *miR-146a* and *miR-146b* target *Traf6* and attenuate TCR signaling in the thymus (53).

FoxP3 is an indispensable transcription factor for the generation and the regulatory function of regulatory T cells. *miR-146a* does not seem critical to the formation of Treg cells in the thymus. Indeed, the role of *miR-146a* has been investigated in FoxP3^+^ Treg cells in the thymus and peripheral lymphoid organs of *miR-146a*-deficient mice. These mice contain significantly increased numbers of FoxP3^+^ Treg cells in the periphery, but not in the thymus (54).

In the human thymus, it was shown that miR-146a and miR-146b are the most up-regulated miRNAs as DP thymocytes differentiate in SP CD4^+^ or CD8^+^ thymocytes (26).

#### miR-155

miR-155 is a highly studied immuno-miR and plays an important role in T-cell homeostasis as fully reviewed by Mashima (55).

In the mouse thymus, the absence of *miR-155* in hematopoietic cells tends to decrease the global representation of thymocytes but it is likely due to a defect occurring at an early stage of thymocyte development, as the proportions of DN, DP, and CD4^+^ and CD8^+^ SP cells are not affected. However, mouse thymic expression of *miR-155* seems to fluctuate in thymocytes at different developmental stages in the thymus (26, 51). In the absence of *miR-155*, a reduction of the number of Treg cells is observed both in the thymus and in the periphery in mice. miR-155 appears to be required for Treg cells development and its absence compromises their proliferation, survival and function in the periphery (56). It was demonstrated that an increased expression of miR-155 in both human and mouse CD4^+^ T helper cells leads to a reduced susceptibility of Treg cells in mediating T-cell suppression, whereas a decreased expression of miR-155- results in a more pronounced suppression by natural Treg cells (57). The control of *miR-155* is also required for the proper development of iNKT cells in the mouse thymus with inhibition of *miR-155* expression along with iNKT cell differentiation (58).

The role of *miR-155* in TECs has not been investigated but its expression decreases with age-induced thymic involution in mice and this might be related to a decreased expression in TECs (29).

#### miR-125

miR-125a and miR-125b sequences are highly conserved throughout diverse species. They have been found differentially regulated in several human disorders (50). They could be involved in inflammatory diseases. miR-125a regulates TNFAIP3 that is a negative regulator of the NF-κB signaling pathway (59). miR-125b targets TRAF6 and regulates IL-1β induced inflammatory genes (60).

*miR-215a* and *miR-125b* are up-regulated in the thymus of aging mice (16, 61) and also in the aging human thymus (Figure 2C). *miR-125a* by targeting *FoxN1*, decreases its expression associated with thymic involution (61). From transcriptomic analyses on purified human or mouse thymocytes and TECs, it was observed that miR-125a and miR-125b are highly expressed in TECs compared to thymocytes (15). Besides, in human thymocytes, miR-125b expression is higher in CD4^+^ as compared to CD8^+^ SP thymocytes (26).

## Pathological Properties of the Thymus From Myasthenia Gravis Patients

Myasthenia Gravis (MG) is an autoimmune disease due to antibodies against several components of the neuromuscular junction. Patients suffer from more or less invalidating muscle weaknesses leading to a generalized fatigability. The majority of patients (85%) displays antibodies against the acetylcholine receptor (AChR). Thymus abnormalities occur in two subtypes of AChR^+^ MG patients, thymoma-associated (TAMG) and AChR^+^ early-onset form (EOMG) (62).

The incidence of TAMG among the MG population is approximately 10-20% (62, 63). Thymomas are rare thymic epithelial cell neoplasms that develop usually in patients after 50 years old, both in women and men. The histological classification by the World Health Organization is based on the nature of the cortical or medullary epithelial cells involved in the tumor: type A for medullary thymoma, type B1 or B2 for respectively predominantly or entirely cortical thymoma, type AB for mixed thymoma (involving both cortical and medullary epithelial cells) and type B3 for cytonuclear atypia thymoma. Several paraneoplastic syndromes are associated with thymoma but the most common is MG with an incidence around 30% but variable from one study to another. Histologically, MG is associated more frequently with type B1, B2, or B3 tumors than with type A or AB tumors. The differences between thymomas and normal thymuses are detailed by Marx and collaborators. Of particular interest neoplastic TECs express less HLA-class II molecules, do not express AIRE in 95% of thymomas and contain reduced numbers of Treg cells (63). These alterations likely promote autoimmune disease occurrence. In addition, the activation of innate immune pathways and an IFN-I signature is observed in thymomas in TAMG (64).

In contrast, EOMG concerns usually female patients before 45–50 years old. Thymus in EOMG is the site of profound structural alterations. One of the main feature characterizing the thymus in AChR^+^ MG is a lymphoproliferative hyperplasia characterized by increased number of B cells and ectopic germinal centers (GCs). The incidence of lymphoproliferative hyperplasia is approximately 70% of patients with AChR^+^ MG, and thymectomy has proven to be efficacious (65). The hyperplastic MG thymus displays all the characteristics of tertiary lymphoid organs. Neoangiogenic processes with high endothelial venule and lymphatic vessel development are clearly observed. Several studies have demonstrated the overexpression or differential expression of chemokines, specifically key molecules involved in peripheral cell recruitment and GC development, such as CXCL13 and CCL21 (66). How the thymus turns into a tertiary lymphoid organ is not well-known but local inflammation seems mandatory. Numerous cytokines are overexpressed in the EOMG thymus. In particular, IFN-β orchestrates thymic changes associated with MG (67, 68) and favors the differentiation of pathogenic Th17 T cells (69). As well, different inflammatory signaling pathways are activated, such as Toll-Like Receptor (TLR) and NF-KB pathways that potentially orchestrate thymic changes.

The hallmarks of disease involve dysfunction of cellular pathways including changes in miRNA expression. In MG differentially expressed miRNAs have been noted in the circulating PBMCs (70–73). The concept of miRNA expression as initiation of disease or the maintenance of specific lymphocyte populations has led to the identification of potential biomarkers. The role of miRNAs in MG thymuses has also been studied as detailed below.

## miRNA Profile in Thymoma of TAMG

Studies to assess the alterations of miRNAs in thymoma are limited. A study performed by Li, et al. on four thymoma samples compared to four control thymus samples demonstrated the differential expression of 137 miRNAs. Further analysis was performed on miR-125a-5p whose expression is increased in TAMG. They identify FoxP3 as one of the targets of miR-125a-5p (74) and FoxP3 expression is known to be altered in thymoma patients (75).

In their study, Li et al. also identified a decreased expression of miR-376a and miR-376c in TAMG patients. These miRNA could be of interest as their expression is decreased in AIRE-silenced TECs (19) and AIRE expression is known to be strongly down-regulated in TAMG (76).

In a separate study, the expression of miR-19b-5p, which targets thymic stromal lymphopoietin (TSLP), was elevated in MG-related thymomas. The negative correlation with TSLP mRNA contributes to T-cell imbalance and promotes MG-related thymoma development (77). Whereas, the levels of miR-20b were significantly decreased compared to those in adjacent non-tumor tissues, resulting in an increased proliferation of T cells through a NFAT5/CAMTA1 dependent pathway (78).

In an independent analysis of miR-150 expression, we observed a significant decreased of this miRNA expression in the thymus of TAMG patients (data not shown). Regarding its high expression in the thymus (Table 1), its role in thymoma should be further investigated.

Interestingly, a miRnome study was led on thymic epithelial tumors, including thymomas but it is not mentioned whether patients had a concomitant autoimmune MG. It could be interesting to know retrospectively which patients with a given miRNA profile later developed MG (79).

## miRNA Profile in the Thymus of EOMG Patients

### Specific Dysregulated miRNAs in EOMG

Three miRnome studies have addressed the changes in miRNA expression in the thymus of EOMG patients (11, 80, 81). The multiple differences in the study designs include the thymus samples from EOMG, the designation of controls, the miRNA arrays, and the method of analysis.

The study by Cron et al. used thymus from untreated female EOMG patients compared to healthy age-matched female controls. The thymus samples from untreated EOMG patients displayed both low hyperplasia with 2 or fewer GCs per section and high hyperplasia with an increased number of GCs. The study was performed using the Affymetrix GeneChip miRNA 3.0 Array. 61 miRNAs are found dysregulated (24 up- and 37 down-regulated). The implication of miRNA clusters localized at the extremity of the X chromosome is also demonstrated. Among the dysregulated miRNAs, they focused their attention on the most down-regulated miRNAs: miR-7-5p. Its down-regulation in MG thymuses is confirmed, in particular in TECs, and an inverse correlation is observed between the expression of miR-7 and CCL21; a target mRNA for miR-7 (11). CCL21 is involved in the abnormal recruitment of B cells in the MG thymus and known to participate in GC formation (82).

As with TAMG studies, miR-125a-5p was elevated in the EOMG thymus samples compared to controls. miR-125a-5p by targeting FoxP3 could explain why the suppressive activity of regulatory T cells is severely impaired in EOMG patients and associated with a decreased expression of FoxP3 in CD4^+^ T cells (83). miR-125a is also known to modulate inflammatory signaling pathways, such as TNFAIP3 (Tumor Necrosis Factor Alpha-Induced Protein 3) a key molecule in the negative regulation of NF-κB (Nuclear Factor κB) and TLR signaling pathways (59, 84), and WDR1 (WD repeat-containing protein 1) implicated in auto-inflammatory processes associated with IL-18 expression (11).

miR-29a is among the miRNAs down-regulated in AIRE^+^ mTECs identified by Ucar et al. (15), and Cron et al. observed that all miRNAs of the miR-29 family were down-regulated in the thymus of EOMG patients (unpublished data). miR-29a is of particular interest regarding its role in IFN-I signaling sensitization (14) and the central role of IFN-β in the EOMG thymus (67).

The study by Li et al. compared thymic miRNA expression in EOMG patients and controls using the Agilent Human miRNA array (V.18.0). This study was performed using four control and four MG thymuses. They extracted 33 dysregulated miRNAs with numerous down-regulated miRNAs from the miR-548 family. Next, they showed that miR-548k targets the 3′ UTR region of CXCL13 decreasing its expression in Jurkat cells (81). Knowing that CXCL13 is up-regulated in MG TECs and participate to B-cell recruitment and GC development (85), this miRNA could thus play a role in MG pathogenesis.

The study by Sengupta et al. used thymus from EOMG patients and separated the samples based on the presentation of GCs (80). For miRNA assessment 13 out of the 16 MG patients were treated with prednisolone (65). The experimental group contained samples with GCs, whereas, the control samples were thymus samples from EOMG patients that did not contain GCs in the thymus block. The Affymetrix GeneChip miRNA 4.0 Array identified 44 mature miRNAs that are altered in GC rich thymus samples from EOMG compared to EOMG patients with no GC expression. The dysregulated expression of 38 miRNAS is confirmed by RT-PCR (8 up- and 30 down-regulated). These miRNAs and targeted mRNAs are involved in regulatory pathways common to inflammation and immune response, cell cycle regulation and anti-apoptotic pathways. The Regulator of G protein Signaling 13 (RGS13), involved in GC regulation, is identified in EOMG thymuses with GCs and was paired with downregulation of miR-452-5p and miR-139-5p. The increased expression of miR-150-5p is found in EOMG samples with GCs which mirrored the miR-150-5p expression in sera of MG patients. miR-150 is also more expressed in the thymus of MG patients compared to healthy controls, and in particular in patients displaying a high degree of thymic hyperplasia (86). *In situ* hybridization analyses showed that miR-150 is strongly expressed by B cells of the mantle zone around GCs. The increased expression of miR-150 in hyperplastic MG thymuses seems thus linked to the abnormal presence of B cells and in particular to the development of GCs. By removal of the thymus, the sera levels of miR-150-5p are reduced (70) but no correlations between the degree of thymic hyperplasia and serum levels in MG patients are observed (86, 87). miR-150 overexpression in MG thymuses is also inversely correlated with the expression of MYB, the most well-known miR-150 mRNA target that displays four binding sites. MYB is a regulator factor essential for hematopoiesis and is highly expressed in the thymus (88). MYB is also characterized as an early regulator of T-cell associated diseases with an altered expression in autoimmune diseases (89). In the MG thymus, miR-150 could be secreted by B cells and alter MYB expression locally and consequently affect T cells, resulting in the significant alterations of the T-cell repertoire seen in MG (90).

miR-145, miR-24, and miR-143 are reduced in GC rich thymus samples as compared to samples with no GCs, and these miRNAs are also reduced in PBMCs of EAMG rats (91). Another group has also observed a decreased expression of miR-143 in the thymus of a MG mouse model in which mice were engrafted in hyperplastic thymic biopsies from EOMG patients and showed a link with CXCL13 expression in thymocytes (92).

Recently, miR-146, one of the most well-known immuno-miR, has been shown to be down-regulated in the thymus of EOMG patient as compared to age-matched donors. The decrease was localized in the thymic stroma and not linked to the presence of GCs. This deficiency was inversely correlated with increased expression of miR-146 targets such as IRAK1, c-REL and ICOS. Interestingly, the expression of miR-146 and these target genes were normalized in EOMG patients under corticosteroid treatment. Altogether, these results suggest that miR-146 could modulate TLR signaling via IRAK1 and cREL and GC formation via ICOS (93). In addition, miR-146 is of great interest in MG as it is known to control the activation of IFN-I signaling pathways (94). The decrease of miR-146 could be linked with the overexpression of IFN-β in MG thymuses (67). In addition a decrease expression of this miRNA could have a strong impact on T-cell differentiation in MG and could be associated with the defective function of Treg cells (83, 95) and/or the increase of pathogenic Th17 cells (69, 96).

### Pathway Analysis of miRNA Expression in EOMG

To progress in the understanding in the implication of miRNAs in EOMG thymuses beyond individual miRNAs, we provide functional relevance of the miRNAs differentially expressed in two miRnome studies (11, 80). Original results from these two publications have been used and miRNA lists were analyzed through miRNet (https://www.mirnet.ca/miRNet/home.xhtml) and targeted genes used to defined pathways. KEGG Pathway enrichment analyses were performed on dysregulated miRNAs identified in Cron et al. (Table 3A) and Sengupta et al. (Table 3B). Despite the difference in these two studies, one comparing thymuses from control donors vs. untreated EOMG patients (11) and the other one comparing thymuses rich in GCs vs. those with no GCs (80), interesting information results from these analyses. Numerous cancer pathways are represented but also pathways related to pathogen infection and TLRs. Pathogens are environmental factor that potentially drive/perpetuate autoimmunity (97) and the thymus is a common target organ for infectious diseases (98). Poliovirus-infected macrophages and the presence of Epstein-Barr virus (EBV)-infected B cells in MG thymus were described in MG thymus (99, 100). Besides, changes regarding TLR expression in MG thymus have been demonstrated (67, 101–103). The chemokine signaling pathway was also found dysregulated in both studies. This is not surprising regarding the role of chemokines in B-cell recruitment and GC development (82, 85, 104). Altogether, these studies provide novel clues to the potential pathways that occur in the hyperplastic thymus and the development of GCs.

**Table 3:** KEGG Pathway enrichment analyses were performed on dysregulated miRNAs identified in Cron et al. **(A)** and Sengupta et al. **(B)** using miRNet (https://www.mirnet.ca/miRNet/home.xhtml).

**Pathway**	**Hits**	***P*-value**
**A**		
Pathways in cancer	90	6.49E-29
Prostate cancer	35	2.74E-15
Focal adhesion	53	1.75E-14
Pancreatic cancer	30	2.21E-14
Glioma	28	2.38E-13
Chronic myeloid leukemia	29	8.4E-13
Colorectal cancer	23	5.21E-12
Small cell lung cancer	29	1.04E-11
Regulation of actin cytoskeleton	44	8.61E-11
Neurotrophin signaling pathway	35	1E-10
Melanoma	25	2.36E-10
Acute myeloid leukemia	22	1.26E-09
Bacterial invasion of epithelial cells	21	6.06E-09
Non-small cell lung cancer	20	8.82E-09
Chemokine signaling pathway	41	1.12E-08
Adherens junction	23	1.49E-08
Renal cell carcinoma	21	2.05E-08
Toxoplasmosis	26	5.40E-08
ErbB signaling pathway	25	5.50E-08
Endometrial cancer	17	1.16E-07
Jak-STAT signaling pathway	26	1.96E-07
HTLV-I infection	39	4.22E-07
T cell receptor signaling pathway	25	6.37E-07
Insulin signaling pathway	30	1.22E-06
Chagas disease (American trypanosomiasis)	23	1.55E-06
p53 signaling pathway	19	4.85E-06
Cholinergic synapse	23	5.12E-06
B cell receptor signaling pathway	20	5.17E-06
Bladder cancer	12	5.17E-06
mTOR signaling pathway	15	5.53E-06
Epstein-Barr virus infection	22	8.07E-06
Hepatitis C	23	1.14E-05
Leukocyte transendothelial migration	24	1.27E-05
MAPK signaling pathway	43	1.39E-05
Apoptosis	20	2.34E-05
Progesterone-mediated oocyte maturation	19	5.02E-05
Type II diabetes mellitus	14	6.24E-05
Fc epsilon RI signaling pathway	18	7.05E-05
Fc gamma R-mediated phagocytosis	21	7.34E-05
Melanogenesis	21	1.38E-04
Thyroid cancer	10	1.50E-04
VEGF signaling pathway	17	2.95E-04
TGF-beta signaling pathway	18	3.21E-04
Hypertrophic cardiomyopathy (HCM)	9	3.30E-04
Axon guidance	22	4.47E-04
Tight junction	22	4.47E-04
Wnt signaling pathway	25	4.97E-04
Tuberculosis	28	7.46E-04
Aldosterone-regulated sodium reabsorption	10	8.17E-04
Carbohydrate digestion and absorption	7	1.11E-03
Alcoholism	26	1.86E-03
Cell cycle	21	2.22E-03
Dopaminergic synapse	21	2.22E-03
Measles	18	3.27E-03
Arrhythmogenic right ventricular cardiomyopathy (ARVC)	5	5.68E-03
ECM-receptor interaction	15	7.47E-03
Osteoclast differentiation	19	7.74E-03
GnRH signaling pathway	16	8.65E-03
Herpes simplex infection	17	8.84E-03
Dilated cardiomyopathy	14	9.31E-03
Adipocytokine signaling pathway	12	1.10E-02
Toll-like receptor signaling pathway	16	1.13E-02
Salmonella infection	13	1.18E-02
Influenza A	16	2.90E-02
Basal cell carcinoma	9	3.19E-02
Cardiac muscle contraction	4	3.59E-02
Dorso-ventral axis formation	4	3.59E-02
NOD-like receptor signaling pathway	9	4.00E-02
Cytokine-cytokine receptor interaction	30	4.15E-02
Viral myocarditis	6	4.15E-02
**B**		
Pathways in cancer	87	3.17E-26
Chronic myeloid leukemia	33	4.40E-16
HTLV-I infection	55	9.17E-16
Prostate cancer	34	1.64E-14
Colorectal cancer	25	6.48E-14
Pancreatic cancer	29	1.88E-13
Focal adhesion	51	2.62E-13
Melanoma	27	6.84E-12
Glioma	26	1.40E-11
Non-small cell lung cancer	22	2.12E-10
Endometrial cancer	20	3.46E-10
Bladder cancer	16	8.40E-10
Small cell lung cancer	26	2.43E-09
Cell cycle	33	2.95E-09
Thyroid cancer	14	6.34E-08
Osteoclast differentiation	30	7.38E-08
ErbB signaling pathway	25	7.70E-08
Renal cell carcinoma	20	1.53E-07
Toll-like receptor signaling pathway	26	1.62E-07
Regulation of actin cytoskeleton	38	1.62E-07
Toxoplasmosis	25	2.78E-07
Acute myeloid leukemia	19	2.92E-07
Adherens junction	21	4.24E-07
Chagas disease (American trypanosomiasis)	24	4.36E-07
p53 signaling pathway	20	1.10E-06
mTOR signaling pathway	16	1.10E-06
Bacterial invasion of epithelial cells	18	1.10E-06
MAPK signaling pathway	46	1.25E-06
Leukocyte transendothelial migration	26	1.25E-06
VEGF signaling pathway	21	1.57E-06
Epstein-Barr virus infection	22	9.71E-06
Neurotrophin signaling pathway	26	1.63E-05
B cell receptor signaling pathway	19	2.30E-05
Axon guidance	25	2.31E-05
TGF-beta signaling pathway	20	3.37E-05
Fc epsilon RI signaling pathway	18	8.65E-05
Apoptosis	19	1.01E-04
T cell receptor signaling pathway	21	1.05E-04
Chemokine signaling pathway	31	3.46E-04
Influenza A	21	3.99E-04
Hepatitis C	20	4.38E-04
Wnt signaling pathway	25	6.63E-04
Measles	19	1.66E-03
Insulin signaling pathway	23	1.95E-03
Salmonella infection	15	1.95E-03
Jak-STAT signaling pathway	18	3.02E-03
Type II diabetes mellitus	11	4.79E-03
Progesterone-mediated oocyte maturation	15	5.83E-03
Epithelial cell signaling in Helicobacter pylori infection	9	8.37E-03
Carbohydrate digestion and absorption	6	8.44E-03
Natural killer cell mediated cytotoxicity	21	1.08E-02
GnRH signaling pathway	16	1.08E-02
Amyotrophic lateral sclerosis (ALS)	9	1.14E-02
Fc gamma R-mediated phagocytosis	16	1.44E-02
Aldosterone-regulated sodium reabsorption	8	1.65E-02
ECM-receptor interaction	14	2.21E-02
Cholinergic synapse	15	2.73E-02
Long-term potentiation	12	3.04E-02
Amoebiasis	9	3.26E-02
Tight junction	17	3.95E-02
Dorso-ventral axis formation	4	3.96E-02
Legionellosis	8	3.96E-02
Hypertrophic cardiomyopathy (HCM)	6	3.96E-02
Arrhythmogenic right ventricular cardiomyopathy (ARVC)	4	3.96E-02
One carbon pool by folate	5	4.62E-02
Viral myocarditis	6	4.62E-02

*Pathways of interest are highlighted: cancer pathways (gray), pathogen infection pathways (blue), Toll-Like Receptor signaling pathway (green) and chemokine signaling pathway (yellow)*.

## Conclusion

Thymic changes occur during life either in the context of thymic involution upon aging /stress or in MG. Here, we review the literature and used original data to describe specific miRNAs that could play a key role in these thymic changes. Among miRNAs that are dysregulated in EOMG or TAMG patients, miR-19b, miR-20b, miR-24, and miR-150 are listed as ThymiRs, and only miR-19b and miR-20b are actively involved in thymic involution. The literature often describes these miRNAs as playing a role in T-cell differentiation. The lack of correlation between miRNAs regulated upon thymic involution, and dysregulated in EOMG or TAMG thymuses suggests the immune response in the autoimmune condition is not directed at the dysregulation of the developmental pathway of the thymus. Most of the down-regulated thymic miRNAs in EOMG are associated with GC development, such as miR-7, miR-24, miR-139, miR-143, miR-145, miR-146, miR-150, miR-452, miR-548, or thymic inflammation, such as miR-125b, miR-146, or miR-29. Further investigations on these miRNAs could now help deciphering if they could represent therapeutic tools to normalized thymic inflammation and associated lymphofollicular hyperplasia in EOMG patients.

## Author Contributions

MC performed the experiments and revised the manuscript. ÉG performed bioinformatics analyses and revised the manuscript. LK and RL wrote the manuscript.

## Conflict of Interest

The authors declare that the research was conducted in the absence of any commercial or financial relationships that could be construed as a potential conflict of interest.

## Abbreviations

AChR: Acetylcholine receptor
AIRE: Autoimmune regulator (AIRE)
DN: Double negative
DP: Double positive
EOMG: Early-onset MG
GC: Germinal center
IFN: Interferon
MG: Myasthenia gravis
miRNA: microRNA
NKT: Natural killer T cell
SP: Simple positive
TAMG: Thymoma-associated MG
TCR: T-cell receptor
TEC: Thymic epithelial cell
cTEC: Cortical thymic epithelial cell
mTEC: Medullary thymic epithelial cell
TLR: Toll-Like Receptor
Treg cell: regulatory T cell
TSA: Tissue-specific antigen.
